# Nomograms Predicting Self-Regulated Learning Levels in Chinese Undergraduate Medical Students

**DOI:** 10.3389/fpsyg.2019.02858

**Published:** 2020-01-15

**Authors:** Jun Yang, Guoyang Zhang, Runzhi Huang, Penghui Yan, Peng Hu, Lanting Huang, Tong Meng, Jie Zhang, Ruilin Liu, Ying Zeng, Chunlan Wei, Huixia Shen, Miao Xuan, Qun Li, Meiqiong Gong, Wenting Chen, Haifeng Chen, Kaiyang Fan, Jing Wu, Zongqiang Huang, Liming Cheng, Wenzhuo Yang

**Affiliations:** ^1^Shanghai Tongji Hospital, Tongji University School of Medicine, Shanghai, China; ^2^Graduate School of Education, Shanghai Jiao Tong University, Shanghai, China; ^3^Division of Spine Surgery, Department of Orthopedics, Tongji Hospital, Tongji University School of Medicine, Shanghai, China; ^4^Key Laboratory of Spine and Spinal Cord Injury Repair and Regeneration (Tongji University), Ministry of Education, Shanghai, China; ^5^The First Affiliated Hospital of Zhengzhou University, Zhengzhou, China; ^6^School of Finance, Henan University of Economics and Law, Zhengzhou, China; ^7^Key Laboratory of Arrhythmias, Ministry of Education, Shanghai East Hospital, Tongji University School of Medicine, Shanghai, China; ^8^Tongji University School of Medicine, Tongji University, Shanghai, China; ^9^Department of Endocrinology, Tongji Hospital, Tongji University School of Medicine, Shanghai, China; ^10^Office of Educational Administration, Naval Medical University, Shanghai, China; ^11^Basic Medical College of Fudan University, Shanghai, China; ^12^Office of Educational Administration, Bengbu Medical College, Bengbu, China

**Keywords:** medical undergraduate, self-regulated learning, multi-center cross-sectional study, nomogram, validation

## Abstract

**Purpose:**

The purpose of this study was to construct a multi-center cross-sectional study to predict self-regulated learning (SRL) levels of Chinese medical undergraduates.

**Methods:**

We selected medical undergraduates by random sampling from five universities in mainland China. The classical regression methods (logistic regression and Lasso regression) and machine learning model were combined to identify the most significant predictors of SRL levels. Nomograms were built based on multivariable models. The accuracy, discrimination, and generalization of our nomograms were evaluated by the receiver operating characteristic curves (ROC) and the calibration curves and a high quality external validation.

**Results:**

There were 2052 medical undergraduates from five universities in mainland China initially. The nomograms constructed based on the non-overfitting multivariable models were verified by internal validation (C-index: learning motivation: 0.736; learning strategy: 0.744) and external validation (C-index: learning motivation: 0.986; learning strategy: 1.000), showing decent prediction accuracy, discrimination, and generalization.

**Conclusion:**

Comprehensive nomograms constructed in this study were useful and convenient tools to evaluate the SRL levels of undergraduate medical students in China.

## Introduction

Self-regulated learners are commonly characterized as active learners, managing their own learning experience in many different kinds of ways (Zimmerman, 1989; Goradia and Bugarcic, 2017). In theory, self-regulated learners own a large arsenal of metacognitive and cognitive strategies that they readily deploy. When it is necessary, they can use them to accomplish academic tasks. Also, self-regulated learners have flexible learning goals and try their best to achieve those goals. Finally, self-regulated students are good at monitoring and modify their strategies in response to the shifting task demands (Zimmerman, 2002; van Houten-Schat et al., 2018). In brief, self-regulated learners are independent, motivated, and metacognitively active participants in their learning process. Self-regulation is one of the most essential skills for medical students (Zimmerman, 1989; Isik et al., 2018).

Prior studies had attached the importance on self-regulation by connecting students characterized as self-regulated learners with positive learning outcomes. Emerging research suggested that SRL had significant predicative effect on medical students’ academic achievement, such as clinical practical skill, innovative ability (White, 2007; Artino et al., 2010). And many factors (parent’s education background, self-efficacy, peers, etc.) influence SRL ability (Turan et al., 2009; Schutz et al., 2011; Brydges and Butler, 2012; Berkhout et al., 2015). However, while a series of studies investigated the current status of SRL ability among specific groups of medical students, there was still no evidences at present describing the SRL levels of Chinese medical undergraduates in more than five centers. Besides, most previous researches focused on descriptive rather than predictive and perspective studies.

What’s more, in current medical studies, nomograms were constructed to predict prognosis of a wide variety of neoplasms and some other diseases, recognized as replaceable or even original criterions compared with traditional staging systems (Balachandran et al., 2015). Therefore, as an accurate prediction tool that could predict the prognosis of severe diseases, the nomogram could also be used to predict SRL level.

Hence, in this study, we performed a multi-center cross-sectional study with a large sample size, identifying the most significant predictors of SRL levels by combining regression analysis methods (logistic regression model and Lasso regression) and machine learning model (random forest), firstly constructing nomograms to predict SRL levels of Chinese medical undergraduates.

## Materials and Methods

### Sample Selection and Data Extraction

This study was approved by the Ethics Committee of Tongji Hospital, Tongji University School of Medicine (No. KYSB-2018-165). This study is designed as a quantitative cross-sectional study conducted in from five universities of mainland China during the academic year 2018–2019.

We selected medical undergraduates by random sampling from five universities in mainland China, including one specialized medical college (Bengbu Medical College), one “project 211” university (Zhengzhou University), two project 985 universities (Fudan University and Tongji University), and one Military Medical University (Naval Medical University). Demographic information, socio-economic status (SES), and SRL score (measured by a professional scale by Zhu et al., 2005) were acquired as the variables in this study. First of all, to evaluate the quality and readability of the questionnaire 20 fourth- and fifth-year students from Tongji University School of Medicine were randomly selected to carry out a pilot study. Questionnaire was modified each student’ comments. After that, the final questionnaire was conducted using the Wenjuanxing website^1^ and the link was sent to the correspondence author of each center. In Bengbu Medical College, Zhengzhou University, Tongji University, and Naval Medical University, the links of the questionnaire were distributed randomly through the medical school administration system to twice the number of students enrolled each year (for example, Tongji University School of Medicine’s annual enrollment was about 125 undergraduates, so the target number of questionnaires in Tongji University School of Medicine was 250). Besides, meanwhile, in the Shanghai Medical College of Fudan University, we randomly sent questionnaires to 40 undergraduates face-to-face on campus. And we excluded students who had unknown and inaccurate variables. At the same time, to eliminate the heterogeneity between universities, a PSM was performed with Tongji University as the reference. Ultimately, we got an initial dataset (without Fudan University) showing no age heterogeneity.

### Statistical Analysis

Before primary statistical analysis, we divided the initial dataset into two cohorts by random sampling as training set and validation set, respectively (sample size: 2:1). Exploratory factor analysis (EPA) was used to determine the score of each students’ SRL scores in the dimensions of motivation and strategy.

The primary data analysis began with descriptive statistics of training set: dichotomous variables were reported as percentages while continuous variables were reported as mean ± SD or median (range). SRL scores were divided into new classification (low or high) following the median value. Subsequently, we performed two statistical methods [univariate logistic regression and random forest (Ntree = 500)] to find the most significant predictors associated with the level of SRL scores in two dimensions. Besides, in random forest algorithm, MDG and OOB error were applied to rank the classification contribution and evaluate the classification accuracy (James et al., 2013).

After these procedures, the best subsets of remarkable factors were selected to enforce the multivariable logistics regression models. Lasso regression was performed to ensure that the multifactor models were not overfitting. (In this study, the Lasso regression was only used to ensure that the reduced multivariable logistic regression models were not overfitting rather than for variable selection and modeling). Eventually, the multivariable models consisting of optimum predictors were constructed. We built the nomograms basing on the multivariable models to predict the probability of low SRL scores. The ROC and the calibration curves were used to assess the accuracy and discrimination of our nomograms. Additionally, to verify the generalization of the nomograms, a high quality external validation was performed using the test set from the Fudan University.

Only two-sided *P*-value < 0.05 was thought to be statistical significance. Excerpt for EPA was performed by SPSS 25.0, all other statistical analysis was enforced with R version 3.5.1 software^2^ (Institute for Statistics and Mathematics, Vienna, Austria) (Package: ggplot2, rms, randomForest, non-random, pROC, glmnet).

## Results

### Sample Selection and Characteristics

**FIGURE 1:**
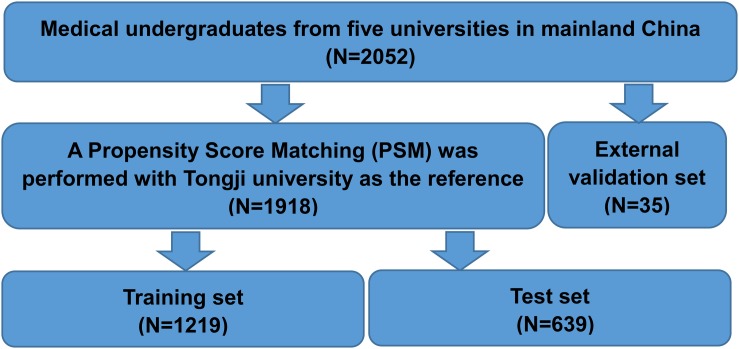
Flow chart for data selection. PSM, propensity score matching.

In this study, 2,440 questionnaires were sent to a corresponding number of undergraduates, 2,111 questionnaires were received, and the results of 2,052 questionnaires could be used for further analysis. The response rate was 84.10%. There were 2,052 medical undergraduates from five universities in mainland China initially. After PSM, we got the dataset of 1,918 students (without Fudan University) showing no age heterogeneity (*P* = 0.265). Then, we divided this dataset into training set (1,219 students) and testing set (639 students) by 2 to 1 by random sampling. The process of data selection is shown by the flow chart in Figure 1.

Each student’s SRL scores in the dimensions of motivation and strategy were determined by EPA. Results of Kaiser–Meyer–Olkin (KMO) test indicated that the sample size were sufficient for EPA (Motivation: *P* = 0.962; Strategy: *P* = 0.984). Table 1 summarizes the SRL status of medical undergraduates in the five universities. After correction, the samples from the five universities showed no demographic heterogeneity (*P* = 0.265). There was no difference in learning motivation (*P* = 0.091) among medical students while significant statistical difference was found in learning strategy (*P* = 0.031). However, we confirmed no significant association between total SRL score and universities (*P* = 0.126).

**TABLE 1:** Self-regulated learning (SRL) status of medical undergraduates in the five universities.

	**Bengbu Medical College**	**The Second Military Medical University**	**Zhengzhou University**	**Fudan University**	**Tongji University**	***P*-value**
Age (years)						0.265
Mean ± SD	20.95 ± 1.82	20.74 ± 1.65	20.86 ± 1.60	20.87 ± 1.59	20.87 ± 1.88	
Median (range)	21 (17–25)	21 (17–25)	21 (17–25)	21 (17–25)	21 (17–25)	
Learning motivation						0.091
Mean ± SD	154.67 ± 16.60	152.73 ± 25.64	154.13 ± 22.84	151.11 ± 28.09	156.48 ± 22.65	
Median (range)	155.53 (105.25–200.74)	153.42 (42.97–211.73)	154.67 (39.30–210.50)	151.18 (53.97–213.61)	157.99 (40.60–209.08)	
Learning strategy						0.031^∗^
Mean ± SD	213.12 ± 30.53	216.75 ± 41.19	212.66 ± 39.24	214.89 ± 48.42	219.50 ± 37.77	
Median (range)	207.54 (114.14–304.42)	210.82 (50.74–304.42)	208.13 (50.74–304.42)	213.13 (50.74–304.42)	220.83 (50.74–304.42)	
Total score						0.126
Mean ± SD	367.79 ± 44.24	369.48 ± 64.82	366.80 ± 59.17	366.00 ± 74.82	375.97 ± 57.91	
Median (range)	361.79 (229.85–492.29)	364.45 (93.78–516.08)	363.48 (90.040–514.93)	356.67 (104.70–518.04)	381.45 (933–513.50)	

*SRL, self-regulated learning; SD, standard deviation. ^∗^*P* < 0.05.*

### Univariate Analysis and Random Forest

Univariate logistic regression and random forest for learning motivation (OOB = 33.46%) and learning strategy (OOB = 31.05%) are shown in Supplementary Table S1. Fifteen variables (GPA, Growing place, Primary caregiver, The person who has the greatest influence on your self-learning, Family economic status, Grade, Does your peer have an impact on your learning, Views on the learning atmosphere of the university, Reasons for choosing medicine, Planning to be a doctor in the future, Time of learning medicine weekly, Time of extracurricular activities weekly, Interest in medicine, Views on the importance of self-learning, and think of the main teaching mode of your university) that not only drew significant results in the univariate logistic regression, but also relative high MDG in the random forest were included in the further multivariable modeling.

### Multivariable Logistic Regression

**FIGURE 2:**
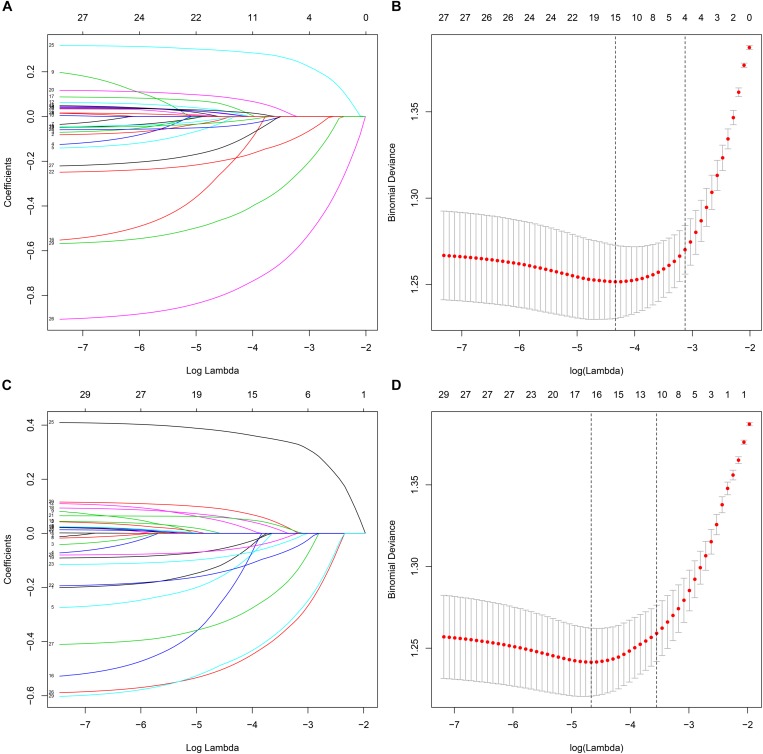
The variables filtering process of the Lasso regression. In order to avoid overfitting, the Lasso regression suggested including 4 and 11 variables when learning motivation level **(A,B)** and learning strategy level **(C,D)** was the endpoint, respectively. In the variable selection process, first of all, the univariate logistic regression and random forest were used to select potential SRL levels indicators. Then, based on these potential indicators, the initial multivariable logistic regression models were constructed. After that, we developed the reduced multivariable logistic regression models by screening the significant variables in the initial models. Additionally, the Lasso regression was performed based on these potential SRL levels indicators found by the univariate logistic regression and random forest. Therefore, in this study, the Lasso regression was only used to ensure that the reduced multivariable logistic regression models were not overfitting rather than for variable selection and modeling.

Fifteen potential significant factors were incorporated into the initial multivariable logistic regression models, and the final multivariable models were constructed to confirm the effects of significant covariates in the initial models to the categorical SRL level (Table 2). All variables eventually incorporated into the multivariate models were shown to be essential to the modeling process in the Lasso regression (Figure 2). Additionally, an analysis of variance (ANOVA) showed no significant difference between initial and final models.

**TABLE 2:** Multivariate logistic regression model for learning motivation levels.

**Variable**	**Learning motivation levels**		**Learning strategy levels**	
		
	**Odds ratio **(95% CI)****	***P*-value**	**Odds ratio **(95% CI)****	***P*-value**
GPA	0.64(0.51,0.80)	< 0.001^∗^	0.60(0.47,0.75)	< 0.001^∗^
**Growing place**				
Rural area	1.00(*reference*)		1.00(*reference*)	
Urban area	0.75(0.57,0.98)	0.033^∗^	0.97(0.74,1.28)	0.835
Urban–rural junction	0.94(0.67,1.33)	0.725	1.05(0.74,1.49)	0.776
**Primary caregiver**				
Father	1.00(*reference*)			
Father and mother	0.48(0.26,0.88)	0.018^∗^		
Mother	0.50(0.24,1.05)	0.066		
Other	0.53(0.23,1.22)	0.134		
**The person who has the greatest influence on your self-learning**				
Companion			1.00(*reference*)	
Father			0.71(0.47,1.07)	0.102
Grandparents			0.40(0.18,0.85)	0.018^∗^
Mother			0.87(0.60,1.28)	0.488
Other relatives			0.44(0.14,1.35)	0.152
Teacher			0.86(0.56,1.34)	0.514
Other			0.71(0.39,1.32)	0.280
**Views on the learning atmosphere of the university**				
Excellent	1.00(*reference*)		1.00(*reference*)	
Good	1.25(0.87,1.79)	0.220	1.25(0.86,1.81)	0.235
Just so-so	1.71(1.18,2.48)	0.005^∗^	1.69(1.16,2.47)	0.007^∗^
Bad	1.31(0.67,2.56)	0.432	1.75(0.87,3.54)	0.117
Terrible	0.97(0.38,2.43)	0.942	1.29(0.49,3.40)	0.607
**Time of learning medicine weekly (h)**				
<10	1.00(*reference*)		1.00(*reference*)	
10–20	0.64(0.45,0.91)	0.014^∗^	0.54(0.38,0.79)	0.001^∗^
20–30	0.49(0.34,0.72)	< 0.001^∗^	0.46(0.31,0.68)	< 0.001^∗^
30–40	0.45(0.29,0.69)	< 0.001^∗^	0.40(0.25,0.63)	< 0.001^∗^
>40	0.20(0.13,0.30)	< 0.001^∗^	0.21(0.14,0.32)	< 0.001^∗^
**Time of extracurricular activities weekly (h)**				
<5			1.00(*reference*)	
5–10			0.64(0.45,0.91)	0.012^∗^
10–15			0.74(0.51,1.09)	0.129
15–20			0.64(0.40,1.02)	0.061
>20			0.69(0.46,1.05)	0.086
**Interest in medicine**				
Extremely interested	1.00(*reference*)		1.00(*reference*)	
Interested	1.40(0.93,2.09)	0.103	2.16(1.40,3.33)	< 0.001^∗^
Just so-so	3.16(2.07,4.83)	< 0.001^∗^	4.45(2.84,6.98)	< 0.001^∗^
Not interested	3.10(1.40,6.85)	0.005^∗^	3.43(1.54,7.68)	0.003^∗^
Extremely not interested	4.00(1.21,13.24)	0.023^∗^	4.95(1.41,17.31)	0.012^∗^
**Think of the main teaching mode of your university**				
Traditional teaching mode			1.00(*reference*)	
Combination of traditional teaching and non-traditional teaching			0.63(0.48,0.83)	0.001^∗^
PBL			0.48(0.28,0.83)	0.008^∗^

*GPA, grade point average; PBL, problem-based learning. ^∗^*P* < 0.05.*

In the final models of training set, low GPA was significantly related to low level of learning motivation (OR, 0.64; 95% CI, 0.51–0.80; *P* < 0.001) and learning strategy (0.60; 0.47–0.75; *P* < 0.001), and parents as primary caregivers were associated with better learning motivation (0.48; 0.26–0.88; *P* = 0.018) than primary caregivers were only fathers. Grandparents as the person who had the greatest influence on your self-learning showed a significant better level in terms of learning strategy (*Companion vs. Grandparents*: 0.40; 0.18–0.85; *P* = 0.018). Furthermore, compared with students who thought the school learning atmosphere was excellent, thinking the learning atmosphere was just so-so was a risk factor of poor learning motivation (1.71; 1.18–2.48; *P* = 0.005) and learning strategy (1.69; 1.16–2.47; *P* = 0.007) levels. Besides, longer time of learning medicine weekly was protective indictor of both learning motivation (*10–20 h*: 0.64; 0.45–0.91; *P* = 0.014; *20–30 h*: 0.49; 0.34–0.72; *P* < 0.001; *30–40 h*: 0.45; 0.29–0.69; *P* < 0.001; *>40 h*: 0.20; 0.13–0.30; *P* < 0.001) and learning strategy (*10–20 h*: 0.54; 0.38–0.79; *P* = 0.001; *20–30 h*: 0.46; 0.31–0.68; *P* < 0.001; *30–40 h*: 0.40; 0.25–0.63; *P* < 0.001; *>40 h*: 0.21; 0.14–0.32; *P* < 0.001) (reference group: <10 h) levels while 5–10 h extracurricular activities weekly was only for better learning strategy (<5 vs. 5–10 h: 0.64; 0.45–0.91; *P* = 0.012). About interest in medicine, extremely interested in medicine was associated with higher levels of learning motivation (*Just so-so*: 3.16; 2.07–4.83; *P* < 0.001; *Not interested*: 3.10; 1.40–6.85; *P* = 0.005; *Extremely not interested*: 4.00; 1.21–13.24; *P* = 0.023) and learning strategy (*Interested*: 2.16; 1.40–3.33; *P* < 0.001; *Just so-so*: 4.45; 2.84–6.98; *P* < 0.001; *Not interested*: 3.43; 1.54–7.68; *P* = 0.003; *Extremely not interested*: 4.95; 1.41–17.31; *P* = 0.012). Additionally, in teaching modes, combination of traditional teaching and non-traditional teaching mode (0.63; 0.48–0.83; *P* = 0.001) and PBL (0.48; 0.28–0.83; *P* = 0.008) were resulted in better learning strategy level than traditional teaching mode.

### Nomogram and Validation

**FIGURE 3:**
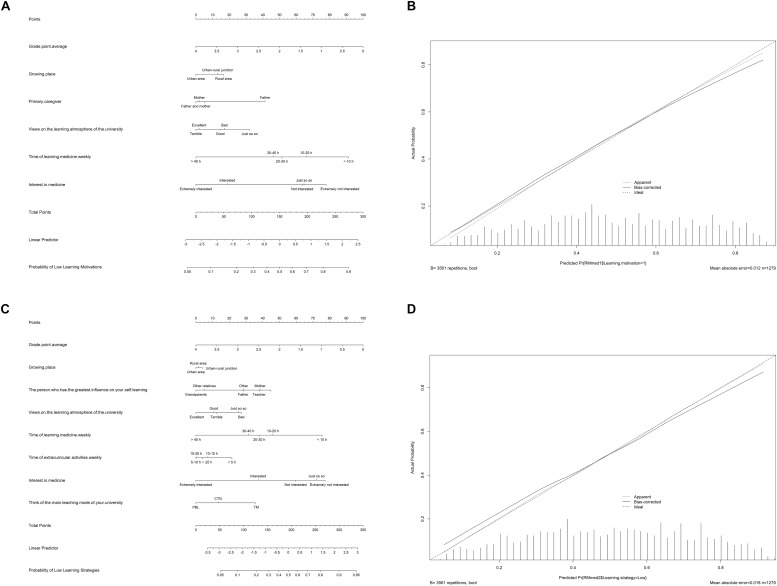
Nomograms and calibration curves of predicting learning motivation level **(A,B)** and learning strategy level **(C,D)**. Based on the final multivariable Logistic regression models, the nomograms predicting learning motivation [area under curve (AUC): 0.733; internal validation C-index, 0.736] and learning strategy level (AUC: 0.749; Internal validation C-index, 0.744) were established, which were defined as the final models predicting SRL levels in Chinese undergraduate medical students. The prediction models could predict SRL levels of the other undergraduate medical students outside the study cohort. For instance, for a senior who just entered the hospital as an intern, the teachers could investigate the variables information in the nomograms of this intern and calculate the possibility of low SRL levels. According to the predicting results, teachers could quantitatively identify students with low SRL levels and conduct personalized intervention. PBL, problem-based learning; TM, traditional teaching mode; CTN, combination of traditional teaching and non-traditional teaching.

Based on the final multivariable logistic regression models, the nomograms predicting learning motivation (AUC: 0.733; internal validation C-index, 0.736) and learning strategy level (AUC: 0.749; internal validation C-index, 0.744) were established, which were defined as the final models predicting SRL levels in Chinese undergraduate medical students (Figures 3A,C and Supplementary Figures S1A–D) (the raw data of training and test dataset are available in the Supplementary Material B). The calibration plots of the CIF are shown in Figures 3B,D, where the points are just slightly farther from the 45-degree line indicating a high goodness of fit between predicted and observed values.

Finally, we carried out an external validation testing 35 undergraduates’ information from the Fudan University (the raw data are available in the Supplementary Material C). The cohort comprised 20 males and 15 females, with a median age of 21 (range, 17–25) years. The mean learning motivation and learning strategy scores were 151.11 ± 28.09 and 214.89 ± 48.42, respectively. And the C-index of external validation (learning motivation: 0.986; learning strategy: 1.000) further illustrated the high prediction accuracy of the nomograms (Supplementary Figures S1E,F).

## Discussion

In brief, self-regulated learners are independent, motivated, and meta-cognitively active participants in their learning process. Self-regulation is one of the most essential skills for medical graduates (Zimmerman, 1989; Berkhout et al., 2015; Goradia and Bugarcic, 2017; van Houten-Schat et al., 2018). Therefore, identifying the vital indicators of SRL levels capacitates timely management to improve the ability of SRL, which may bring about better education outcomes and less cost. Currently, we performed a multi-center cross-sectional study with a large sample size, identifying the most significant predictors of SRL levels by combining regression analysis methods and machine learning model, firstly constructing and validating nomograms to predict SRL levels of Chinese medical undergraduates. Our results suggested that our prediction models had good performance, whether discrimination, calibration, accuracy, or generalization.

### Current Situation of Chinese Medical Undergraduates’ SRL Levels

In our series, after PSM, the total cohorts included 1,209 males and 790 females, with a median age of 21 (range, 17–25) years old, similar to previous studies (Artino et al., 2010; Brydges and Butler, 2012; Berkhout et al., 2015; Jouhari et al., 2016; van Houten-Schat et al., 2018). Due to some special features of medicine (e.g., differences male–female ratio, size of each grade in different schools), age was used as the matching variable in the use of PSM to eliminate inter-group heterogeneity.

In summary, the SRL ability of Chinese medical undergraduates was common. The average score of learning motivation was slightly higher than the median while the average score of learning strategy was slightly lower. Consistent with previous studies, the SRL levels of medical students in learning motivation and learning strategy were generally not high, and there was no significant difference between majors and schools (Isik et al., 2018; Visser et al., 2018). In China, this might be attributable to the subjective intention rather than passive adjustment of the most medical students applying for the medicine, so their learning motivation is slightly higher. However, due to the lack of emphasis on the cultivation of SRL ability in most Chinese universities, traditional teaching mode, and individual psychological quality of students, the learning strategy level of medical students was generally not high as students in other majors (Brydges and Butler, 2012; Berkhout et al., 2015; Isik et al., 2018).

### Factors Influencing SRL Levels of Undergraduate Medical Students in China

With the combination of machine learning model and classic regression analysis methods, “GPA,” “views on the learning atmosphere of the university,” “time of learning medicine weekly,” and “interest in medicine” were identified demonstrated to be independent indicators for both learning motivation levels and learning strategy levels in Chinese medical undergraduates. “Growing place” and “primary caregiver” only significantly influenced learning motivation levels while the same results were achieved for “The person who has the greatest influence on your self-learning,” “time of extracurricular activities weekly,” and “think of the main teaching mode of your university” for learning strategy levels.

#### Learning Environment and Outcomes (Teaching Mode and GPA)

In this study, GPA was demonstrated to be an independent predictive factor for SRL level and the higher the GPA, the stronger the SRL ability, similar to some previous studies (Yip, 2009; Schutz et al., 2011; Jouhari et al., 2016). Students with high GPA had greater motivation and more effective learning strategies than other students, inclined to invest more time in medical learning and have more interest in medicine.

At the same time, the traditional teaching mode also had a significant negative impact on the learning strategy levels. Traditional teacher-centered teaching was not conducive to the cultivation of students’ active thinking and SRL ability. Numerous previous studies had found that PBL made students self-centered and self-leading, while teachers became mentors to manage and guide the learning process (Brummer, 2004). And more recently, the practice-based learning and improvement (PBLI) also highlighted the interaction between students and learning context (Pringle and Lee, 1998; Brummer, 2004; Mahajan et al., 2017). These new teaching modes could fully mobilize students’ learning enthusiasm, and stimulate their interest in learning so that they were more willing to invest more time and energy in learning (Artino et al., 2010; Berkhout et al., 2015; van Houten-Schat et al., 2018). Additionally, the subgroup analysis revealed that students who spend more time in studying were inclined to get higher GPA (Supplementary Table S2).

Therefore, improving students’ teaching modes and curriculum setting could provide students with more judicious learning strategies, benefiting to students’ better academic performance, and so on in a virtuous circle.

#### Time Investment (Time of Learning Medicine and Extracurricular Activities Weekly)

The results of this study firstly suggested that longer medical study time and appropriate extracurricular activities (5–10 h per week) were the momentous predictors associated with SRL levels. Previous studies had shown that medical students with strong learning motivation tend to be interested in medicine and willing to spend more time in medical studies (Berkhout et al., 2015; Isik et al., 2018). which was also in line with the results of our subgroup analysis (Supplementary Table S3, students who were not interested in medicine tended to spend less time studying medicine). Thus, students insisting on continuous learning had higher SRL levels.

Surprisingly, 5–10 h of extracurricular activities per week was a protective factor for low learning strategy level in the multivariable model. Many studies accentuated the importance of extracurricular activities for (pre)-medical students. Stress and burnout, mainly due to heavy schoolwork, progressively developed over the course of medical education (Fares et al., 2016). Appropriate extracurricular activities could reduce anxiety, stress, and burnout and their harmful effects on physical and mental health (Stewart et al., 1997; Almalki et al., 2017; Urlings-Strop et al., 2017). However, our results confirmed that excessive extracurricular activities (>10 h per week) were not positive to SRL level. Hence, it was more essential to maintain a balance between schoolwork and extracurricular activities, which might be the vital element of the learning strategy (Slavin, 2016; Almasry et al., 2017).

#### Social Psychological Environment (Growing Place, Primary Caregiver, the Person Who Has the Greatest Influence on Your Self-Learning, Views on the Learning Atmosphere of the University, and Interest in Medicine)

In this study, we confirmed that social psychological environment of medical students was critical to SRL. Compared with students in cities, students in rural area had significant lower learning motivation levels. This might be related to parents’ education level, less stressful living environment, peers, and so on. Besides, it had been reported that the SRL ability of medical students was significantly correlated with the academic, social, psychological, and educational environment (Brydges and Butler, 2012; Berkhout et al., 2015; Slavin, 2016; van Houten-Schat et al., 2018). Medical science is a discipline integrating natural science and social science. Due to the particularity of the clinical environment, the SRL in the clinical environment was affected by the specific goals, experiences, and learning opportunities, all of which varied with the individual, environmental, and social attributes (Slavin, 2016; van Houten-Schat et al., 2018).

Therefore, medical students’ SRL were more susceptible to the influence of social psychological environment than students of other majors.

### Nomograms

Comprehensive nomograms constructed in this study were useful and convenient tools to evaluate the SRL levels of undergraduate medical students in China. The nomograms were verified by internal validation (C-index: learning motivation: 0.736; learning strategy: 0.744) and external validation (C-index: learning motivation: 0.986; learning strategy: 1.000), showing decent prediction accuracy, discrimination, and generalization (Balachandran et al., 2015). To our best knowledge, it was the first time to establish nomograms predicting the SRL levels of medical students. With the help of the nomograms, teachers could accurately identify potential students with low SRL level and intervene in time according to the risk factors.

However, there were some limitations that need to be addressed. First, because the initial cohort in this study contained data from multiple centers, its inter-group heterogeneity could not be completely eliminated, even though we had a strict criterion to minimize this heterogeneity. Secondly, limited by sample size, the C-index of external validation was relatively high, suggesting that large sample size study was still needed. Last but not least, random sampling had some systematic errors and cross-sectional study had the weaknesses thereof. Nonetheless, notwithstanding these limitations, this study did construct first nomograms which could well predict the SRL levels of undergraduate medical students in China.

In the future, we expected to provide feedbacks for multiple stakeholders to improve the education quality of medical students. Our subsequent researches will expand the sample size, externally validate and correct the nomograms, and further carry out prospective intervention studies for the vital predictors.

## Conclusion

Despite its limitations, this study did construct first nomograms which could well predict the SRL levels of undergraduate medical students in China. In the future, more comprehensive prediction models should be designed to achieve higher accuracy and generalization. Subsequent researches should focus on large sample size prospective intervention studies, validating and correcting the nomograms.

## Data Availability Statement

All datasets generated for this study are included in the article/Supplementary Material.

## Ethics Statement

This study was approved by the Ethics Committee of Tongji Hospital, Tongji University School of Medicine (No. KYSB-2018-165).

## Author Contributions

JY, GZ, RH, WC, HC, KF, JW, ZH, LC, and WY: conception and design, and manuscript writing. WC, HC, KF, JW, ZH, LC, and WY: provision of study material and collection and/or assembly of data. JY, GZ, RH, PY, PH, LH, TM, JZ, RL, YZ, CW, HS, MX, QL, and MG: data analysis and interpretation. JY, GZ, RH, PY, PH, LH, TM, JZ, RL, YZ, CW, HS, MX, QL, MG, WC, HC, KF, JW, ZH, LC, and WY: final approval of manuscript.

## Conflict of Interest

The authors declare that the research was conducted in the absence of any commercial or financial relationships that could be construed as a potential conflict of interest.

## Footnotes

1 https://www.wjx.cn

2 www.r-project.org

## Abbreviations

AUC: area under curve
CI: confidence interval
GPA: grade point average
MDG: mean decrease Gini
OOB: out-of-bag
OR: odds ratio
PBL: problem-based learning
PSM: propensity score matching
ROC: receiver operating characteristic curve
SD: standard deviation
SES: socioeconomic status
SRL: self-regulated learning.
