# Supplementary material for: Nomograms Predicting Self-Regulated Learning Levels in Chinese Undergraduate Medical Students
Source: Front Psychol. 2020 Jan 15;10:2858. doi: 10.3389/fpsyg.2019.02858 (PMC6974523; doi:10.3389/fpsyg.2019.02858)
Supplement: Supplementary file 1 [file Data_Sheet_1.docx]

Supplementary Material

Supplementary materials A:

Table S1 shows univariate logistic regression and random forest for learning motivation and learning strategy.

Table S2 – S3 shows the subgroup univariate analysis results.

Figure S1 shows the receiver operating characteristic curves (ROCs) evaluating the accuracy of training dataset modeling (A, B), internal validation (C, D) and external validation (E, F).

Supplementary materials B:

The raw dataset of the initial dataset after PSM consisted of 1918 samples.

Supplementary materials C:

The raw dataset of the validated set consisted of 35 samples from Fudan university.

**Table S1** Results of single factor analysis and random forest in training dataset

| Variables | Learning motivation | | Learning strategy | |
| --- | --- | --- | --- | --- |
|  | P value of logistic regression | MDG | P value of logistic regression | MDG |
| **Age** | 0.338 | 29.97 | 0.420 | 31.05 |
| **Grade point average** | < 0.001* | 59.81 | < 0.001* | 62.29 |
| **Gender** |  | 8.60 |  | 9.00 |
| Female | Reference |  | Reference |  |
| Male | 0.612 |  | 0.087 |  |
| **Growing place** |  | 16.54 |  | 15.35 |
| Rural area | Reference |  | Reference |  |
| Urban area | 0.018* |  | 0.532 |  |
| Urban-rural junction | 0.794 |  | 0.256 |  |
| **Only child** |  | 8.47 |  | 8.73 |
| No | Reference |  | Reference |  |
| Yes | 0.510 |  | 0.427 |  |
| **Family structure** |  | 6.88 |  | 6.38 |
| One-parent family | Reference |  | Reference |  |
| Parental family | 0.801 |  | 0.946 |  |
| Reorganized family | 0.464 |  | 0.253 |  |
| **Primary caregiver** |  | 13.64 |  | 12.68 |
| Father | Reference |  | Reference |  |
| Father and mother | 0.028* |  | 0.220 |  |
| Mother | 0.031* |  | 0.097 |  |
| Other | 0.283 |  | 0.155 |  |
| **Father's education level** |  | 16.79 |  | 17.47 |
| Bachelor | Reference |  | Reference |  |
| College degree | 0.481 |  | 0.831 |  |
| Doctor and above | 0.211 |  | 0.354 |  |
| Junior high school and below | 0.177 |  | 0.461 |  |
| Master | 0.782 |  | 0.845 |  |
| **Father's occupation** |  | 7.32 |  | 7.26 |
| Occupation mainly based on manual labor | Reference |  | Reference |  |
| Occupation requiring professional skills and mental work | 0.561 |  | 0.578 |  |
| **Mother's education level** |  | 15.99 |  | 15.97 |
| Bachelor | Reference |  | Reference |  |
| College degree | 0.253 |  | 0.075 |  |
| Doctor and above | 0.208 |  | 0.087 |  |
| Junior high school and below | 0.189 |  | 0.131 |  |
| Master | 0.618 |  | 0.884 |  |
| **Mother's occupation** |  | 6.96 |  | 6.96 |
| Occupation mainly based on manual labor | Reference |  | Reference |  |
| Occupation requiring professional skills and mental work | 0.491 |  | 0.343 |  |
| **The type of guardians' learning support and interventions** |  | 23.91 |  | 24.20 |
| Accompanying | Reference |  | Reference |  |
| Indulgent | 0.088 |  | 0.196 |  |
| Problem-intervening | 0.700 |  | 0.349 |  |
| Strictly managed | 0.954 |  | 0.758 |  |
| **The person who has the greatest influence on your self-learning** |  | 37.20 |  | 36.49 |
| Companion | Reference |  | Reference |  |
| Father | 0.074 |  | 0.010* |  |
| Grandparents | 0.315 |  | 0.004* |  |
| Mother | 0.392 |  | 0.248 |  |
| Other relatives | 0.822 |  | 0.175 |  |
| Teacher | 0.679 |  | 0.589 |  |
| Other | 0.770 |  | 0.254 |  |
| **Family economic status** |  | 16.45 |  | 17.36 |
| Average | Reference |  | Reference |  |
| Poor | 0.234 |  | 0.192 |  |
| Very poor | 0.273 |  | 0.162 |  |
| Good | 0.029* |  | 0.157 |  |
| Very good | 0.455 |  | 0.735 |  |
| **Family monthly income (RMB)** |  | 20.51 |  | 20.44 |
| < 5000 | Reference |  | Reference |  |
| 5000-10000 | 0.739 |  | 0.847 |  |
| 10000-15000 | 0.249 |  | 0.494 |  |
| > 15000 | 0.062 |  | 0.057 |  |
| **Native place of origin** |  | 38.69 |  | 40.58 |
| China Central | Reference |  | Reference |  |
| China Northeast | 0.686 |  | 0.916 |  |
| China East | 0.345 |  | 0.120 |  |
| China South | 0.592 |  | 0.251 |  |
| China North | 0.735 |  | 0.610 |  |
| China Northwest | 0.523 |  | 0.993 |  |
| China Southwest | 0.185 |  | 0.904 |  |
| **University** |  | 20.00 |  | 19.01 |
| Bengbu Medical College | Reference |  | Reference |  |
| Tongji University | 0.399 |  | 0.060 |  |
| Zhengzhou University | 0.915 |  | 0.586 |  |
| The Second Military Medical University | 0.878 |  | 0.177 |  |
| **Major** |  | 4.04 |  | 4.00 |
| Clinic Medicine | Reference |  | Reference |  |
| Other | 0.480 |  | 0.541 |  |
| **School system** |  | 12.43 |  | 12.15 |
| Five years | Reference |  | Reference |  |
| Seven years | 0.251 |  | 0.283 |  |
| Eight years | 0.886 |  | 0.401 |  |
| Ten years | 0.607 |  | 0.061 |  |
| **Grade** |  | 28.19 |  | 29.01 |
| Fifth grade | Reference |  | Reference |  |
| First grade | 0.314 |  | 0.523 |  |
| Second grade | 0.894 |  | 0.606 |  |
| Third grade | 0.702 |  | 0.094 |  |
| Fourth grade | 0.170 |  | 0.006* |  |
| **Does your peer have an impact on your learning?** |  | 13.23 |  | 13.64 |
| No | Reference |  | Reference |  |
| Yes, positive | 0.006* |  | 0.002* |  |
| Yes, negative | 0.144 |  | 0.343 |  |
| **Views on the learning atmosphere of the university** |  | 25.11 |  | 25.24 |
| Excellent | Reference |  | Reference |  |
| Good | 0.056 |  | 0.031* |  |
| Just so so | < 0.001* |  | < 0.001* |  |
| Bad | 0.076 |  | 0.004* |  |
| Terrible | 0.180 |  | 0.033* |  |
| **Reasons for choosing medicine** |  | 16.74 |  | 18.70 |
| Interested in medicine | Reference |  | Reference |  |
| Parental coercion | < 0.001* |  | < 0.001* |  |
| Other | < 0.001* |  | < 0.001* |  |
| **Planning to be a doctor in the future** |  | 16.96 |  | 16.77 |
| Have no decision | Reference |  | Reference |  |
| No | 0.211 |  | 0.440 |  |
| Yes | < 0.001* |  | < 0.001* |  |
| **Time of learning medicine weekly** |  | 54.13 |  | 53.37 |
| < 10 h | Reference |  | Reference |  |
| 10-20 h | < 0.001* |  | < 0.001* |  |
| 20-30 h | < 0.001* |  | < 0.001* |  |
| 30-40 h | < 0.001* |  | < 0.001* |  |
| > 40 h | < 0.001* |  | < 0.001* |  |
| **Time of extracurricular activities weekly** |  | 35.74 |  | 36.47 |
| < 5 h | Reference |  | Reference |  |
| 5-10 h | 0.015* |  | 0.002* |  |
| 10-15 h | 0.720 |  | 0.103 |  |
| 15-20 h | 0.065 |  | 0.021* |  |
| > 20 h | 0.627 |  | 0.289 |  |
| **Interest in medicine** |  | 36.11 |  | 38.48 |
| Extremely interested | Reference |  | Reference |  |
| Interested | 0.001* |  | < 0.001* |  |
| Just so so | < 0.001* |  | < 0.001* |  |
| Not interested | < 0.001* |  | < 0.001* |  |
| Extremely not interested | 0.001* |  | < 0.001* |  |
| **Views on the importance of self-learning** |  | 31.08 |  | 20.48 |
| Extremely unimportant | Reference |  | Reference |  |
| Unimportant | 0.598 |  | 0.417 |  |
| Have no decision | 0.160 |  | 0.712 |  |
| Important | 0.981 |  | 0.330 |  |
| Indispensable | 0.014* |  | 0.034* |  |
| **Think of the main teaching mode of your university** |  | 13.27 |  | 15.47 |
| Traditional teaching mode | Reference |  | Reference |  |
| Combination of traditional teaching and non-traditional teaching | 0.056 |  | < 0.001* |  |
| PBL | 0.024* |  | < 0.001* |  |

**NOTE:** Categorical variables were compared by using the Pearson Chi-square test. Continuous variables in normal distribution and homogeneity of variance were compared by using the two-sample t test, otherwise, the Mann-Whitney U test were performed.

**Abbreviations:** GPA, Grade Point Average; RMB, RenMinBi; PBL, Problem-Based Learning.

* P < 0.05.

**Table S2** Subgroup analysis results between GPA and time of learning medicine weekly

| Time of learning medicine weekly | OR | 95%CI | P value |
| --- | --- | --- | --- |
| < 10 h | 1.00(reference) |  |  |
| 10-20 h | 1.15 | 1.05-1.25 | 0.003* |
| 20-30 h | 1.25 | 1.14-1.37 | < 0.001* |
| 30-40 h | 1.21 | 1.09-1.35 | < 0.001* |
| > 40 h | 1.43 | 1.29-1.57 | < 0.001* |

**Note:** Time of learning medicine weekly < 10 h is the reference group.

**Abbreviations:** OR, odd ratio; CI, confidence interval; GPA, Grade Point Average.

*P < 0.05

**Table S3** Subgroup analysis results between time of learning medicine weekly and interest in medicine

| Time of learning medicine weekly | interest in medicine | OR | 95%CI | P value |
| --- | --- | --- | --- | --- |
| ≤ 20 h | Extremely interested | 1.00(reference) |  |  |
|  | Interested | 1.54 | 1.07-2.20 | 0.019* |
|  | Just so-so | 3.22 | 2.21-4.68 | < 0.001* |
|  | Not interested | 4.87 | 2.35-10.11 | < 0.001* |
|  | Extremely not interested | 7.10 | 2.21-22.79 | < 0.001* |

**Note:** Time of learning medicine weekly > 40 h is the reference group.

**Abbreviations:** OR, odd ratio; CI, confidence interval.

*P < 0.05


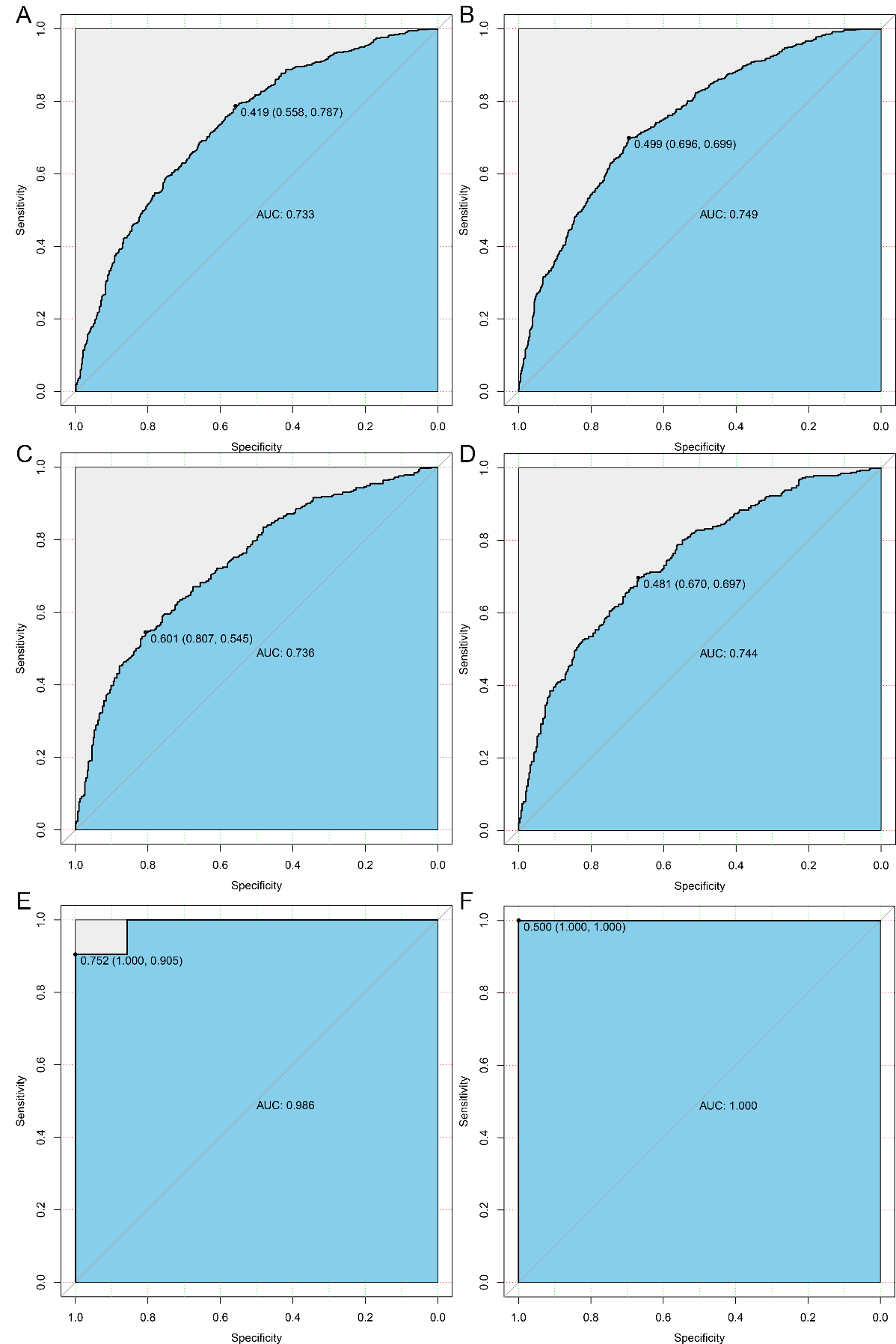


**Supplementary Figure 1**. The receiver operating characteristic curves (ROCs) evaluating the accuracy of training dataset modeling (A, B), internal validation (C, D) and external validation (E, F).
